# Impact of Primary Care Mental Health Management on Emergency Psychiatric Presentations: A Systematic Review

**DOI:** 10.7759/cureus.99601

**Published:** 2025-12-19

**Authors:** Anas E Ahmed, Rahf A Hakami, Atyaf M Alrajhi, Bayan A Buhulaigah, Laura M Damanhouri, Wejdan A Majrashi, Hanouf H Alhamyani, Lujain A Alamer, Asim H Alanazi, Fatimah H Fageehi

**Affiliations:** 1 Community Medicine, Jazan University, Jazan, SAU; 2 College of Medicine, Jazan University, Jazan, SAU; 3 College of Medicine and Medical Sciences, Arabian Gulf University, Manama, SAU; 4 College of Medicine and Surgery, King Abdulaziz University, Jeddah, SAU; 5 College of Medicine, King Abdulaziz University, Jeddah, SAU; 6 College of Medicine, Taif University, Taif, SAU; 7 General Practice, King Faisal University, Al-Hassa, SAU; 8 General Practice, University of Tabuk, Tabuk, SAU; 9 General Practice, Jazan University, Jazan, SAU

**Keywords:** behavioral health homes, collaborative care, continuity of care, emergency department utilization, mental health integration, primary care, psychiatric emergencies, service utilization

## Abstract

Primary care-based mental health management has been proposed as a strategy to reduce preventable psychiatric crises and lessen reliance on emergency departments, yet its impact remains uncertain across different populations and health systems. This review synthesized evidence from observational and quasi-experimental studies evaluating integrated, collaborative, or behavioral health home interventions within primary care and their association with emergency psychiatric utilization. Nine eligible studies were identified from a comprehensive search, most involving co-located or integrated behavioral health services aimed at improving continuity and outpatient engagement. Overall, interventions demonstrated consistent trends toward fewer psychiatric emergency visits, enhanced follow-up care, and better support for individuals with serious mental illness, although effect sizes varied and several studies were limited by methodological weaknesses such as confounding and selection bias. Two higher-quality studies showed moderate reductions in all-cause and psychiatric emergency use, while others reported mixed or modest effects. Collectively, the findings suggest that integrated primary care mental health approaches hold promise for reducing emergency presentations, but stronger, well-controlled research is needed to clarify which models yield the most meaningful and sustained reductions in acute care utilization.

## Introduction and background

Mental health-related emergency department (ED) visits have increased substantially worldwide. This trend places growing pressure on acute care systems. Individuals experiencing crises related to depression, anxiety, psychosis, substance use, or suicidal ideation often turn to the ED. Fragmented outpatient services and limited community mental health capacity contribute to this pattern [1-3]. The rising demand leads to ED overcrowding, prolonged wait times, psychiatric boarding, higher healthcare costs, and disrupted continuity of care [1-3]. Reducing preventable psychiatric ED presentations has become a priority for health systems and policymakers [1-3].

Primary care plays a critical role in the early detection and ongoing management of mental health conditions. It is the most accessible point of contact for many individuals with emerging or chronic symptoms [3-5]. However, traditional primary care models often face challenges. These include limited mental health expertise, insufficient collaboration with behavioral health specialists, and structural barriers that hinder proactive follow-up [3-5]. Such limitations can create gaps in care, increasing the likelihood of clinical deterioration and crisis-driven ED use [3-5]. Strengthening mental health capacity within primary care is a key strategy for improving outcomes and reducing unnecessary reliance on acute services [1-5].

Integrated primary care mental health models, including patient-centered medical homes (PCMHs), primary care behavioral health (PCBH) models, behavioral health homes, and various co-located or collaborative approaches, have gained traction as potential solutions [1-6]. These models commonly incorporate embedded mental health professionals, structured care coordination, shared electronic health records, routine screening, and systematic follow-up [1-6]. By enhancing continuity and supporting earlier intervention, integrated care aims to prevent escalation of symptoms that lead to ED presentations [1-6]. Emerging evidence suggests that such models may reduce psychiatric ED visits and improve psychosocial outcomes, although findings vary across settings and populations [1-9].

Despite growing interest in integration, the literature assessing its direct impact on psychiatric ED utilization remains fragmented and methodologically diverse. Studies differ in their definitions of integration, the populations they evaluate, and the outcomes they measure [1-9]. Some report meaningful reductions in ED use, while others find modest or inconsistent effects [1-9]. Individuals with serious mental illness represent a particularly high-risk population, yet research focused on this group remains limited and often susceptible to confounding [2,6,8,9]. These gaps highlight the need for a systematic synthesis of existing evidence [1-9].

This systematic review aims to evaluate the impact of primary care mental health management on reducing psychiatric ED presentations across diverse integration models and healthcare environments [1-9]. By clarifying the effectiveness of these interventions, the review seeks to inform policy, guide resource allocation, and support the development of more effective pathways that reduce crisis-driven utilization of ED services for individuals with mental health conditions [1-9].

## Review

Methods

Literature Search Strategy

A systematic search was conducted in PubMed, the Cochrane Library, Web of Science, and Scopus to identify studies evaluating primary care mental health integration and its impact on ED psychiatric or behavioral health presentations. Searches covered all publication years through December 2025 and used combinations of subject headings and free-text terms related to primary care models, mental health conditions, emergency services, and healthcare utilization. Search strategies were adapted for each database. Only English-language human studies using observational or quasi-experimental designs were included.

Eligibility Criteria

Eligibility was defined using the Population-Exposure-Comparator-Outcome (PECO) framework [10]. Eligible studies examined adults or mixed-age populations with mental health conditions receiving services in primary care settings, or individuals with psychiatric or behavioral health-related ED presentations. Exposures included any model of integrated or enhanced primary care mental health-such as co-located behavioral health services, collaborative care, the PCMH model, the PCBH model, behavioral health homes, or similar interventions. Eligible studies used a comparator group or a pre-post design and reported at least one ED-related outcome, including total ED visits, psychiatric ED visits, frequent ED use, ED-to-primary care ratios, or psychiatric admissions. Exclusion criteria included pediatric-only studies, ED-based interventions lacking a primary care component, non-original research, case reports, editorials, non-English publications, conference abstracts, and studies without ED utilization outcomes.

Study Selection

All records were imported into a reference management system, and duplicates were removed. Two reviewers independently screened titles and abstracts, excluding studies that did not meet population, exposure, design, or outcome criteria. Full texts of potentially eligible studies were reviewed by both reviewers, with disagreements resolved through discussion or a third reviewer. The screening process followed Preferred Reporting Items for Systematic Reviews and Meta-Analyses (PRISMA) guidelines and is summarized in the PRISMA flow diagram [11].

Data Extraction and Quality Appraisal

Two reviewers independently extracted study characteristics, including setting, design, sample demographics, type of integrated care model, comparator group, and ED-related outcomes such as total visits, psychiatric visits, and high-utilization patterns. Disagreements were resolved by consensus. Risk of bias was assessed using the Risk of Bias in Non-randomized Studies of Interventions (ROBINS-I) tool across domains, including confounding, participant selection, intervention classification, deviations from intended interventions, missing data, outcome measurement, and selective reporting [12]. Most studies showed moderate to serious risk of bias due to observational designs, baseline imbalances, self-selection, and reliance on administrative datasets. These assessments informed the narrative synthesis.

Results

Study Selection

**Figure 1:**
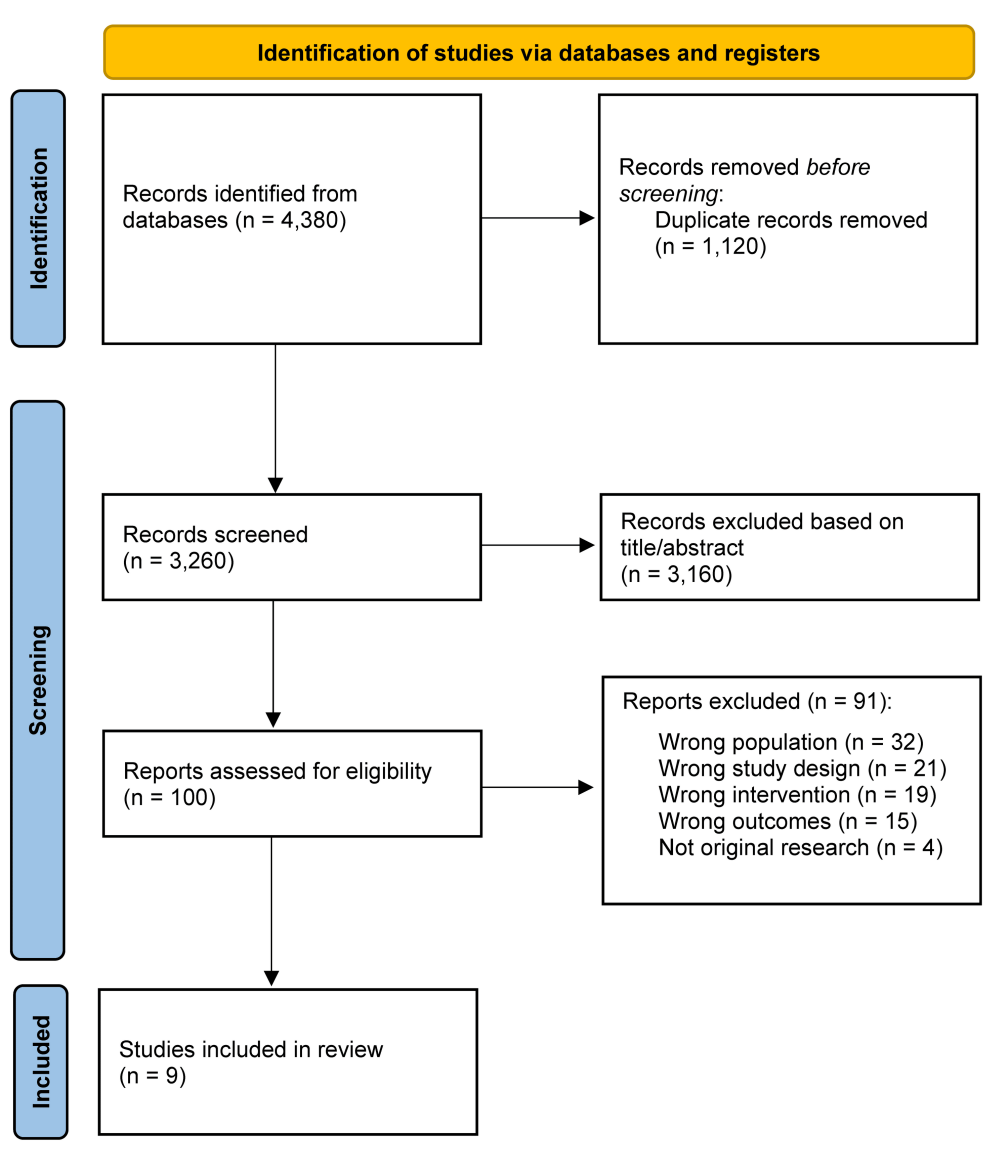
PRISMA flow diagram PRISMA: Preferred Reporting Items for Systematic Reviews and Meta-Analyses

The search yielded 4,380 records from PubMed (n = 1,432), Scopus (n = 1,534), Web of Science (n = 884), and the Cochrane Library (n = 530). After removing duplicates, 3,260 records were screened, with 3,160 excluded due to irrelevance, lack of ED outcomes, non-psychiatric focus, or non-original study design. One hundred full texts were reviewed, and 91 were excluded for reasons including wrong population, wrong design, wrong intervention type, or absence of ED-related outcomes. Nine studies [1-9] met all inclusion criteria and were included for qualitative synthesis. None were suitable for meta-analysis due to substantial heterogeneity. The selection process is summarized in the PRISMA diagram (Figure 1).

Baseline Characteristics

The nine included studies represented diverse U.S. healthcare environments, including safety-net systems and community mental health centers [2-4], urban primary care clinics [7], tertiary hospital EDs [5], PCMHs [1,8], and statewide behavioral health home programs [9]. Most studies used retrospective or quasi-experimental designs, including pre-post comparisons, difference-in-differences analyses, and marginal structural models. Sample sizes ranged from small cohorts of approximately 100 adults with serious mental illness [8] to Medicaid datasets exceeding 12,000 individuals [9]. Populations commonly included adults with serious mental illness, multimorbidity, or high ED utilization (Table 1).

**Table 1:** Characteristics and key findings of the included studies This table summarizes studies evaluating the impact of various integrated care models on ED utilization and inpatient service use among adults with behavioral health conditions across different clinical settings. It includes information on study design, population characteristics, intervention type, comparators, primary outcomes, and key effects on ED and inpatient utilization. Study designs include retrospective cohort studies, quasi-experimental designs, pre-post comparisons, and DID analyses, some combined with propensity score matching to reduce confounding. The interventions represented include PCMHs, IBH, PBHCI, PCBH, and BHH within PRPs. Clinical settings include CMHCs and programs using EHRs or EMRs. Additional outcome categories include ACS conditions. ED: emergency department; PCMH: Patient-Centered Medical Home; IBH: integrated behavioral health; EHR: electronic health record; PBHCI: Primary and Behavioral Health Care Integration; SMI: serious mental illness; BH: behavioral health; PCBH: Primary Care Behavioral Health; PRP: Psychiatric Rehabilitation Program; BHH: Behavioral Health Home; CMHC: community mental health center; EMR: electronic medical record; ACS: ambulatory care-sensitive; DID: Difference-in-Differences

Study ID	Country/Setting	Study Design	Population	Intervention Type	Intervention Description	Comparator	Primary Outcomes	Effect on ED Utilization	Effect on Inpatient Use	Key Findings	Conclusion
Serrano et al. [1]	USA - 4 primary care medical homes, Dane Co., WI	Retrospective quasi-experimental pre-post	Adults ≥18 with ≥1 primary care visit linked to mood/anxiety disorders; N=10,150.	PCBH	BHCs embedded in primary care; brief consults; shared EMR; 1-4 visits/year	One non-integrated clinic	Total ED visits; ED/PC ratio	Wingra clinic: ED/PC ratio ↓ −0.11; other clinics: trend improvement	Not assessed	High baseline ED rates; post-PCBH: leveling/downward trends	PCBH may reduce ED use in some clinics; effects vary by site
Waters et al. [2]	USA - CMHC with Integrated Health Clinic	Retrospective pre-post	N=370 adults with SMI; ≥6 months pre/post enrollment	Integrated Primary Care within CMHC	Physical health services inside CMHC; co-located teams; separate EMRs	Pre-post	Physical health ED, inpatient admissions/days; ACS conditions	ED visits ↑ 0.073 → 0.603; OR 10.15	Inpatient admissions/days ↑	Very low chronic disease utilization; data gaps possible	CMHC integrated care did not reduce ED/inpatient use; trends increased; findings limited by low comorbidity capture
Breslau et al. [3]	USA - 7 NYC CMHCs (PBHCI)	Quasi-experimental; DID with propensity score	Adults 18-64 with SMI; Wave 1: N=6,716 PBHCI, 13,039 controls; Wave 2: N=1,887 PBHCI, 11,542 controls	PBHCI	Primary care inside mental health clinics; chronic disease monitoring; wellness programs	Specialty mental health clinics without integration	(1) ED visits; (2) Frequent ED (≥4/yr); (3) Inpatient stays; (4) Frequent inpatient (≥3/yr)	Wave 1: Behavioral-health ED visits ↓ (OR=0.89); Wave 2: no effect	Medical inpatient stays ↑ (Wave1 OR=1.21; Wave2 OR=1.33)	Integration uncovered unmet medical needs; reduction in BH ED use only in Wave 1	PBHCI increases medical admissions but modestly reduces BH ED visits; overall mixed effects
Krupski et al. [4]	USA - 2 safety-net CMHCs, King Co., WA	Retrospective cohort study; DID with propensity score matching	Adults with SMI; Clinic1: N=373; Clinic2: N=389; matched controls	PBHCOn-site	The integrated primary care; nurse care managers; wellness programs; referrals	Matched non-integrated mental health patients	(1) ED visits/costs; (2) Inpatient admissions/costs; (3) Outpatient medical visits/costs	No significant difference	Clinic1: inpatient ↓18%→12%; Clinic2: no change	Outpatient utilization ↑: early integration sites may uncover unmet needs	Mature integration reduces inpatient admissions; ED use remains unchanged; integration is promising, but takes time
Adaji et al. [5]	USA - Mayo Clinic ED; PCMH primary care system	Retrospective cohort (2 yrs)	Behavioral health ED patients; N=3,815; 5,398 ED visits; age 4-93	PCMH	Integrated primary care with collocated IBH team, shared EHR, care coordination, and follow-up calls	Non-PCMH patients	(1) Hospital admission; (2) 72-hr ED revisit	Hospital admission reduced (OR=0.83, 95% CI 0.74-0.93); no effect on 72-hr returns	Less likely to be hospitalized	Strong integration may reduce admissions and improve continuity	PCMH reduces hospitalizations for BH ED patients; no effect on 72-hr revisits
Breslau et al. [6]	USA - PBHCI clinics, 3 states	Multistate quasi-experimental; matched comparison	Adults with SMI and/or co-occurring substance use; >30 PBHCI clinics	PBHCI	Integration of primary care and preventive services; nurse management; chronic disease monitoring; team-based care	Matched non-PBHCI clinics	ED utilization, inpatient stays, Medicaid costs, and preventive care quality	Frequent ED use ↓ (mainly physical health)	Inpatient stays for physical health ↓; hospitalization costs ↓	PBHCI increases outpatient care and preventive service use; variable chronic disease monitoring	PBHCI reduces ED/inpatient use for physical health, lowers costs; preventive care improvements are inconsistent
Chen et al. [7]	USA - Urban medical center, Queens	Retrospective pre-post (2 yrs)	Older adults ≥50; N=920 intervention, N=341 comparison; low-income, racially diverse	Low-intensity integrated primary care + BH	45-min integrated visit: psychiatric screening, lifestyle counseling, med review, referral support	Non-intervention patients	(1) ED use; (2) Cost per visit	ED visits decreased 5.66% → 3.01% of visit-days (p<0.001)	Not assessed	Cost per visit decreased; effect consistent across subgroups	Minimal BH integration within primary care reduces ED use and costs
Belson et al. [8]	USA - UNC WakeBrook Primary Care, NC	Retrospective cohort	N=101 SMI adults with ≥1 ED visit; subset N=50 with chronic comorbidity	Enhanced PC Model for SMI (PCMH-based)	Co-located PCMH; broad primary care; behavioral counseling; peer support; chronic disease monitoring	Pre- and post- within the same patients	Annual ED visits/person	Physical-health ED visits ↓ (Years 3-4: 3.23→1.83; Rate Ratio=0.57)	Not assessed	High baseline ED; decline required ≥3 yrs stable care	Enhanced PC integrated with BH reduces ED use after long-term engagement
Bandara et al. [9]	USA - Maryland Medicaid; BHH in PRPs	Longitudinal cohort; marginal structural modeling	N=12,232 adults with SMI; 3,319 BHH, 8,913 non-BHH	Behavioral Health Home (BHH)	Care management, physical health coordination, social support	PRP patients not enrolled in BHH	(1) All-cause ED; (2) Physical-health ED; (3) BH ED; (4) Inpatient admissions	All-cause ED probability ↓0.26→0.23; driven by physical-health ED ↓	No effect on inpatient admissions	Longer BHH exposure strengthened ED reduction; BHH inpatients may need extra coordination.	BHH slightly reduces ED visits but not admissions; suggests improved physical health coordination

Integrated care models varied widely. Some studies evaluated comprehensive behavioral health integration within mental health settings using the Primary and Behavioral Health Care Integration (PBHCI) model [2-4,6], while others examined behavioral health integration within primary care via the PCMH framework [1,5,8] or the PCBH model using embedded behavioral health consultants [1]. Low-intensity models included screening and referral interventions [7], while enhanced primary care programs located on psychiatric campuses provided intensive chronic disease management [8]. Comparator groups included usual care clinics, non-integrated programs, or pre-intervention periods. Outcomes included ED visit frequency (psychiatric, behavioral, medical, or all-cause), return visits, high ED use, primary care engagement, inpatient admissions, and healthcare costs.

Quality Assessment

Risk of bias was generally serious due to limitations of observational designs. Confounding was the most common concern, as most studies lacked randomization and showed baseline imbalances or self-selection into integrated programs. Serious confounding was noted in Adaji et al. [5], Breslau et al. [3], Chen et al. [7], and Serrano et al. [1]. Selection bias was typically moderate because clinic assignment or participation was voluntary. Intervention classification and deviations from intended interventions were generally low risk (Table 2).

**Table 2:** Quality appraisal summary of the included studies This table presents the ROBINS-I assessment for included studies evaluating integrated care models for adults with behavioral health conditions. Domains assessed include confounding, selection bias, classification of the intervention, deviations from intended interventions, missing data, outcome measurement, and selective reporting. Each study is assigned an overall ROBINS-I risk rating based on domain-level judgments. ROBINS-I: Risk Of Bias In Non-randomized Studies of Interventions; PCMH: Patient-Centered Medical Home; MH: mental health; DID: difference-in-differences; EHR: electronic health record; PCBH: Primary Care Behavioral Health; PBHCI: Primary and Behavioral Health Care Integration; SMI: serious mental illness; MSM: marginal structural modeling; UNCWPC: University of North Carolina WakeBrook Primary Care; CMHC: community mental health center

Study ID	Confounding	Selection Bias	Classification of Intervention	Deviations From Intended Intervention	Missing Data	Outcome Measurement	Selective Reporting	Overall ROBINS-I Risk
Serrano et al. [1]	Serious - quasi-experimental design with partial adjustment	Moderate - clinic assignment	Low	Low	Moderate	Low	Moderate	Serious risk of bias
Waters et al. [2]	Serious - high confounding; no control; self-selection	High	Low	Low	High - missing claims data	Moderate	High	Critical risk of bias
Breslau et al. [3]	Serious - DID + propensity methods used, but waves differ; residual confounding remains	Moderate - clinic-level assignment	Low	Low	Moderate	Low	Moderate	Serious risk of bias
Krupski et al. [4]	Moderate to Serious - DID + matching helps, but baseline SMI severity differences remain	Moderate	Low	Low	Moderate	Low	Low	Serious risk of bias
Adaji et al. [5]	Serious - baseline imbalance; non-random PCMH membership; residual confounding	Moderate - PCMH membership self-selected	Low - PCMH classification clear	Low - no active deviations	Moderate - incomplete reporting of missingness	Low - objective EHR outcomes	Moderate - limited outcomes reported	Serious risk of bias
Breslau et al. [6]	Moderate - large dataset and matched groups, yet confounding still likely	Moderate	Low	Low	Moderate	Low	Low	Moderate risk of bias
Chen et al. [7]	Serious - no control group; pre-post only	Moderate - voluntary participation	Low	Low	Moderate	Low	Serious - outcomes limited to available billing data	Serious risk of bias
Belson et al. [8]	Serious - no control; long pre-post design; multi-year lag	Moderate	Low	Low	Moderate-High	Low	Serious - no protocol and selective analyses	Serious risk of bias
Bandara et al. [9]	Moderate - MSM weighting strong but residual confounding possible	Low-Moderate	Low	Low	Low-Moderate	Low	Low	Moderate risk of bias

Missing data and selective reporting contributed to further concerns. Some studies lacked detailed reporting on missingness [5], while others relied on administrative datasets with limited variables [7,8]. Outcome measurement was generally reliable due to the use of electronic health records or claims data, but showed moderate concerns in Waters et al. [2] due to incomplete data linkage. Selective reporting was graded moderate to serious in studies lacking predefined protocols. Overall, most studies were judged at serious risk of bias, while Bandara et al. [9] and Breslau et al. [6] showed moderate overall risk.

Synthesis of Findings

Across the nine studies, the impact of primary care mental health integration on ED utilization varied widely, reflecting differences in program intensity, patient characteristics, and implementation quality. Three overarching patterns were observed.

First, the maturity and depth of integration strongly influenced outcomes. High-intensity, well-established models such as PCMH-based integrated care [5], PBHCI programs [4], and statewide health homes [9] generally reported reductions in psychiatric or all-cause ED visits. PBHCI programs showed early reductions in behavioral ED visits [3], and statewide health homes improved care coordination, reducing ED reliance [9]. In contrast, early-stage, low-intensity, or poorly implemented integration models showed limited or adverse effects. PCBH implementation reduced ED visits only in clinics with strong organizational readiness [1], while early-phase PBHCI programs increased ED and inpatient visits [2].

Second, patient complexity and unmet medical needs modified the impact of integrated care. In some settings, ED visits increased initially because integrated care uncovered unaddressed medical conditions. PBHCI models reported higher medical inpatient admissions across multiple waves [3], and the Enhanced Primary Care model showed delayed reductions in ED use following sustained engagement [8]. Similarly, Waters et al. [2] observed increased ED visits likely due to newly identified medical conditions. Conversely, integrated care was most effective for patients with complex multimorbidity. Chen et al. [7] and Bandara et al. [9] found greater reductions among individuals with multiple chronic medical conditions. These findings indicate that integrated care is particularly effective when programs are equipped to manage both physical and behavioral health needs.

Third, multiple mechanisms explained reductions in ED use in mature integrated programs. Improved continuity of care, supported by shared electronic records, alert systems, and structured follow-up, helped prevent crises and reduced psychiatric ED recidivism [5,6]. Enhanced access to medical and behavioral health services within the same clinic allowed earlier intervention and improved chronic disease management, contributing to reductions in physical ED visits [9]. Team-based care coordination enabled providers to jointly address medical, psychiatric, and psychosocial drivers of ED use, particularly in clinics with cohesive workflows [1]. Stabilizing high utilizers through consistent follow-up further reduced ED reliance [8]. When these mechanisms were weak or inconsistently implemented, ED use remained unchanged or increased, as seen in Waters et al. [2].

Limitations

This systematic review has several limitations that should be considered when interpreting the findings. First, the included studies were predominantly observational and quasi-experimental in design, which introduces a moderate to serious risk of bias due to confounding, selection bias, and unmeasured differences between intervention and comparator groups. Second, the heterogeneity of integrated care models, populations, settings, and outcome measures limited the ability to perform a quantitative meta-analysis and may reduce the generalizability of results. Third, most studies were conducted in the United States, often within specific healthcare systems such as Medicaid, community mental health centers, or academic medical centers, which may limit applicability to other countries or healthcare contexts. Fourth, several studies relied on administrative data, electronic health records, or claims data, which may underreport clinical nuances, mental health severity, or social determinants affecting ED utilization. Fifth, publication bias is possible, as studies reporting significant reductions in ED use may be more likely to be published than those with null or negative findings. Finally, the review was restricted to English-language publications, which could exclude relevant research from non-English-speaking regions.

## Conclusions

This review indicates that primary care-based mental health management can help reduce psychiatric emergency presentations by improving access to timely care, enhancing symptom stabilization, and supporting continuity across services. Integrated models generally demonstrate greater benefit than coordinated or co-located approaches, although outcomes may vary in settings with limited resources or workforce constraints. Reducing psychiatric crises and easing ED burden requires not only strong primary care integration but also broader system-level support, including sufficient staffing and expanded community mental health capacity. When effectively designed and implemented, integrated primary care can play a critical role in reducing emergency psychiatric utilization.

## References

[1] Serrano N, Prince R, Fondow M, Kushner K (2018). Does the primary care behavioral health model reduce emergency department visits?. Health Serv Res.

[2] Waters HC, Furukawa MF, Jorissen SL (2018). Evaluating the impact of integrated care on service utilization in serious mental illness. Community Ment Health J.

[3] Breslau J, Leckman-Westin E, Han B (2018). Impact of a mental health based primary care program on emergency department visits and inpatient stays. Gen Hosp Psychiatry.

[4] Krupski A, West II, Scharf DM (2016). Integrating primary care into community mental health centers: Impact on utilization and costs of health care. Psychiatr Serv.

[5] Adaji A, Melin GJ, Campbell RL, Lohse CM, Westphal JJ, Katzelnick DJ (2018). Patient-centered medical home membership is associated with decreased hospital admissions for emergency department behavioral health patients. Popul Health Manag.

[6] Breslau J, Sorbero MJ, Kusuke D (2021). Primary and behavioral health care integration program: impacts on health care utilization, cost, and quality. Rand Health Q.

[7] Chen D, Torstrick AM, Crupi R, Schwartz JE, Frankel I, Brondolo E (2019). Reducing emergency department visits among older adults: a demonstration project evaluation of a low-intensity integrated care model. J Integr Care.

[8] Belson C, Sheitman B, Steiner B (2020). The effects of an enhanced primary care model for patients with serious mental illness on emergency department utilization. Community Ment Health J.

[9] Bandara SN, Kennedy-Hendricks A, Stuart EA (2020). The effects of the Maryland Medicaid Health Home Waiver on emergency department and inpatient utilization among individuals with serious mental illness. Gen Hosp Psychiatry.

[10] Schardt C, Adams MB, Owens T, Keitz S, Fontelo P (2007). Utilization of the PICO framework to improve searching PubMed for clinical questions. BMC Med Inform Decis Mak.

[11] Page MJ, McKenzie JE, Bossuyt PM (2021). The PRISMA 2020 statement: an updated guideline for reporting systematic reviews. BMJ.

[12] Sterne JA, Hernán MA, Reeves BC (2016). ROBINS-I: a tool for assessing risk of bias in non-randomised studies of interventions. BMJ.

